# Harvest: an open-source tool for the validation and improvement of peptide identification metrics and fragmentation exploration

**DOI:** 10.1186/1471-2105-11-448

**Published:** 2010-09-06

**Authors:** Leo C McHugh, Jonathan W Arthur

**Affiliations:** 1 Discipline of Medicine, Sydney Medical School, University of Sydney, Sydney, Australia

## Abstract

**Background:**

Protein identification using mass spectrometry is an important tool in many areas of the life sciences, and in proteomics research in particular. Increasing the number of proteins correctly identified is dependent on the ability to include new knowledge about the mass spectrometry fragmentation process, into computational algorithms designed to separate true matches of peptides to unidentified mass spectra from spurious matches. This discrimination is achieved by computing a function of the various features of the potential match between the observed and theoretical spectra to give a numerical approximation of their similarity. It is these underlying "metrics" that determine the ability of a protein identification package to maximise correct identifications while limiting false discovery rates. There is currently no software available specifically for the simple implementation and analysis of arbitrary novel metrics for peptide matching and for the exploration of fragmentation patterns for a given dataset.

**Results:**

We present Harvest: an open source software tool for analysing fragmentation patterns and assessing the power of a new piece of information about the MS/MS fragmentation process to more clearly differentiate between correct and random peptide assignments. We demonstrate this functionality using data metrics derived from the properties of individual datasets in a peptide identification context. Using Harvest, we demonstrate how the development of such metrics may improve correct peptide assignment confidence in the context of a high-throughput proteomics experiment and characterise properties of peptide fragmentation.

**Conclusions:**

Harvest provides a simple framework in C++ for analysing and prototyping metrics for peptide matching, the core of the protein identification problem. It is not a protein identification package and answers a different research question to packages such as Sequest, Mascot, X!Tandem, and other protein identification packages. It does not aim to maximise the number of assigned peptides from a set of unknown spectra, but instead provides a method by which researchers can explore fragmentation properties and assess the power of novel metrics for peptide matching in the context of a given experiment. Metrics developed using Harvest may then become candidates for later integration into protein identification packages.

## Background

Protein identification using mass spectrometry is one of the fundamental tools of proteomics. Liquid Chromatography coupled to Electro-Spray Injection Tandem Mass Spectrometry (LC/MS/MS) [1] is the method of choice for the fully automated, high throughput experiments that increasingly typify proteomics research [2-4]. These methods have pre-processing steps that may include extracting the proteins, digesting the proteins with a cleavage enzyme such as trypsin, and separating the resultant peptides using reverse phase LC columns. The samples are then introduced into the mass spectrometer by means of elution through a nano-spray injector. The output is a large number of complex mass spectra. The challenge of protein identification is to unambiguously match a single theoretical peptide to each of the unidentified spectra resulting from a true peptide fragmentation inside the machine. A confounding factor is the large number of spectra generated from noise, contaminants, and non-peptide products, as well as spectra that do originate from peptides, but are of such low quality that there is not enough reliable information to declare a theoretical match.

These comparisons between the observed and theoretical spectra are implemented as "metrics", which is a mathematical term for a function defining a distance between elements of a set. In the context of peptide metrics, a metric gives a measure of similarity between an observed spectrum and a theoretically generated spectrum. A metric may be a single feature, such as the number of matching peaks between the observed and theoretical spectrum, or it may be a complex function derived from many attributes of the potential match, such a dot product between a theoretically predicted and observed spectrum after mapping each into vectors. A peptide identification metric takes, as input, a candidate theoretical peptide sequence and an observed spectrum, and outputs a score representing the measure of confidence of a match between the two. A perfect metric, when given an unidentified spectrum and a set of candidate theoretical sequences would in every case give the highest score to the correct assignment, and lower scores to all other (incorrect) peptide sequence assignments. In the case where the correct peptide is not among the candidates, the metric would output a score for all candidates below some threshold. In practice, any good metric will produce a distribution of scores for the candidate sequences, with the single correct assignment likely to be amongst the top candidates. Metrics looking at different aspects of the confidence of a match between a candidate sequence and an observed spectrum will produce different distributions for the candidate scores. Well-known examples of metrics in the protein identification context are the Xcorr and S scores in Sequest [5] or the P values in Mascot [6].

Each unidentified spectrum generated from a peptide fragmented inside the mass spectrometer is unique. In theory, every unidentified spectrum that has not been drawn from pure noise should yield enough information to identify it, except for a narrow set of exceptions [4], although in practice the presence of noise and poor quality spectra make many spectra unidentifiable. The fact that a vast number of observed spectra remain unidentified during the protein identification process [7-14] is a consequence of one of two limitations:

1. Poor quality spectra: where the signal peaks are few or difficult to distinguish from the noise.

2. The inability to recognise and exploit enough features of the spectrum to declare a match *i.e.*, the existing metrics are insufficient to perform the task of identification.

It is possible to develop a metric to assign a numerical value representing the similarity between two spectra for any feature against which it is possible to compare those spectra[15]. If additional features of the spectrum can be recognised, or existing features better understood, they can be harnessed to create new or better metrics [16-19].

In this work, we aim to provide a tool for the collection and analysis of data for a number of parameters related to fragmentation spectra. Once this new knowledge is determined for a specific dataset, it can be used to create better metrics for identification algorithms. This is the fundamental approach behind fragmentation machine learning models shown to improve protein identification rates [15-17,20]. Metrics giving a better separation between correct and random peptide assignments show that the new knowledge about the fragmentation process is genuine, and that the metric embodying that knowledge produces a measurable improvement when implemented. Being assured of these two properties of the metric gives the developer a high degree of confidence that the use of the metric will improve the power of a protein identification algorithm.

In order to develop and test these metrics, we required a framework dedicated to the identification of peptides and flexible enough to handle arbitrary new information. We chose for the underlying method a probability approach for two reasons: it is a well established framework for noisy and complex data [16,17] and because many protein identification algorithms rely on probability methods [20-25], so new or better metrics developed using a probability framework can be easily implemented in this class of protein identification algorithms to improve their performance.

In this paper, we demonstrate the collection of dataset specific information about fragmentation spectra and examples of how to embed this knowledge into a probability metric. Then we show how Harvest can be used to validate an improvement in the number and confidence of peptide identifications using this new metric using a subset of the entire dataset. Once validated using Harvest, such a new metric can be used to improve the performance of any of the popular probability based protein identification algorithms, and to validate hypotheses about the poorly understood fragmentation process.

## Implementation

Harvest has been written in C++ using Microsoft Visual Studio v6.0. The Parameter class in the code gives access to all parameters required for testing. The section in which new metrics should be included is clearly marked in the Candidate class definition. Information relating to matching peaks and LOD scores for each dataset is output by default in the working directory, and can be piped to an appropriate file by the user. The LOD score is the log of the odds score, defined as the logarithm of the ratio of the probability the match is correct and the probability the match is random.

Harvest is not a protein identification package. However, in order to compare peptide matching metrics Harvest performs some of the same functions used in protein identification algorithms. It does this to produce a set of high confidence peptide assignments. The set of high confidence assignments can be examined to explore fragmentation properties or to trial the new metric against the dataset so as to measure any improvement in the confidence of peptide assignments or in the number of peptides identified when using the metric under development. The importance of Harvest is the fragment-level reporting, comparison, manipulation, and analysis of novel peptide matching scores through a metric under development, thereby answering hypotheses about fragmentation and applying this information in the form of better peptide identification metrics. This research question does not necessitate globally maximising the number of identified peptides and therefore distinguishes Harvest from existing algorithms that aim to do this, such as protein identification packages, or algorithms operating on the results of such packages.

### Datasets

We used the data from Cooksey *et al*. [26], downloaded in July 2010 from the PRIDE repository http://www.ebi.ac.uk/pride and the Aurum Dataset composed of known proteins [27], as our reference MS/MS datasets. The Aurum dataset is a MALDI produced dataset and contains only +1 precursor ions. The Cooksey dataset was selected because it is publicly available and is broadly typical of a high throughput MS/MS experiment using human tissue and is of a moderate size (77,779 spectra). The Aurum dataset was chosen because it is derived from a known set of 246 input proteins, and therefore can be used to validate the Harvest algorithm. For the Cooksey dataset, the original sample was human plasma, depleted of the most abundant proteins, and cleaved with trypsin. LC analysis was accomplished by strong-cation exchange (SCX) followed by reverse-phase (RP) LC coupled directly in line with ESI IT MS.

The files were unpacked from tar.gz format and converted from mzXML into DTA format using the Peak List Conversion Utility available at Proteome Commons [28] to create folders of DTA format files. This dataset we now refer to as the Cooksey dataset. For the Aurum Dataset, the first 500 identified peptides were individually downloaded from the Aurum manuscript files and used as a reference set.

### Pre-processing and modifications

A program written in C++ and bundled with Harvest provides the necessary pre-processing tools for Harvest. This program needs to be run once only, prior to the use of Harvest, for each new or updated target protein database. It takes a FASTA format file containing the entire proteome of the target organism and produces a binary output file containing peptide objects with pre-computed attributes such as parent mass, modifications, theoretical ion series spectra, an identifier for the protein from which it comes, etc. The file of peptide objects is ordered by mass. The processed human IPI database takes a few seconds to load into memory on a dual core 2.0 MHz Intel machine, and is typically in the order of 500 MB for a tryptic digest of the human proteome with up to 2 missed cleavages. This large size is due to the additional pre-computed information such as theoretical spectra included in the binary object file. However, the use of these pre-processed objects greatly improves the run-time speed of Harvest.

Modifications are dealt with in the Harvest code. A number of common modifications are included by default (oxidation and methylation).The user is able to create new modifications and control the type (fixed/variable) and maximum number of each modification allowed per peptide.

### Load-in

The dataset of unidentified spectra in DTA format is loaded into the application from a directory specified by the user. During this process, a noise level threshold of 0.5% of the total sum of peak intensities was applied to each spectrum to filter out very low intensity peaks. This is consistent with signal processing algorithms previously described [29,30]. In brief, thresholds based on the sum of peak intensities, rather than the intensity of the most intense peak in the spectrum, are used. This is because the intensities of the peaks in the spectrum are relative to the proportion of fragmentation at each site along the length of the *entire *peptide. This means that the properties of the noise are dependent on the peptide, not the most intense fragment. After loading in the input DTA files, the pre-processed peptide objects from the protein database are loaded into memory in a mass ordered array.

### Candidate selection based on precursor mass and number of y ion series matches

This first level of candidate selection in Harvest is similar to that of most common protein identification algorithms [1-5]. Theoretical peptide candidates are selected based on their parent mass falling within a given mass range of the unidentified spectrum parent mass. For each theoretical peptide in the candidate list, the y ion series (stored as part of the peptide object) is compared to the unidentified spectrum. The number of peaks aligned within the specified mass tolerance is recorded. Candidates with the minimum specified number of y hits show initial evidence of being potentially correct assignments and are retained as the candidate set.

### Preliminary scoring

Preliminary scoring is now applied to the candidate set. The candidate set is assumed to contain the correct peptide assignment. Although the candidate set is a tiny subset of the entire theoretical peptide database, it is still large enough so that the application of arbitrarily complex metrics cannot be applied to the set to distinguish the correct assignment due to computational constraints. For this reason, a preliminary scoring stage is used to reduce the candidate set by rejecting candidates showing significant evidence of being a random match. This candidate set reduction stage strikes a balance between speed and accuracy, but is biased heavily towards retaining the correct assignment. It does this by retaining in the candidate set any peptides with even weak evidence of being the correct match. The inclusive nature of this filter is necessary to retain the correct peptide in the candidate set given the expected inaccuracies of such a computationally "cheap" filter.

In Harvest, the preliminary scoring:

• orders the most intense peaks in the unidentified spectrum

• selects the top 4N peaks, where N is the sequence length determined by assuming an average amino acid mass of 114 Da. This step ensures that approximately the same proportion of the most intense peaks with respect to peptide length is used during scoring. Limiting the number of peaks used for scoring is common for candidate selection procedures in protein identification algorithms [5,6,24,31] and improves run-time speed. 4N is chosen as a number that will likely include most of the basic y and b ions, plus a number of other identifiable ions such as iminium (immonium) ions or those generated from the loss of water or ammonia from b or y ions. This choice is arbitrary and a simple percentage of the total intensity or a percentage of the highest intensity peak may be used as a cut-off if desired. If fewer than 4N peaks exist, then all peaks are used.

• For the first 4N peaks:

○ records the sum of intensities for peaks matching iminium, y, or b ions from the theoretical candidate sequence.

○ Records the total sum of intensities of peaks not matched to iminium, y, or b ions.

For the preliminary score, theoretical y1+, b1+, and iminium ions are generated. y2+ ions are also generated if the parent charge is 2 or higher.

The preliminary score is defined as the proportion of intensities matched to these theoretical ions over the sum of all intensities for the first 4N peaks. For example, a preliminary score of 0.5 would result if half of the total intensity of the top 4N peaks is accounted for by peaks matching iminium, y, and b ions. Candidates with a preliminary score below a user-defined threshold are rejected. A low minimum preliminary score accepts more early stage candidates and therefore is more likely to capture the correct peptide assignment, while a high peptide score reduces the number of candidates and therefore reduces run-times. The default for a minimum preliminary candidate score is 0.05, as this score was found sufficient to ensure the capture of high confidence peptide assignments across a range of datasets. If during the use of Harvest a user finds consistently that high confidence assignments have preliminary scores above a certain threshold, they may increase the minimum preliminary score to this value to speed run times. For example, a value of 0.30 was found to maintain sensitivity without sacrificing specificity for some datasets (especially MALDI).

### Probability scoring

At this final stage there are few enough candidates to allow significant computational effort to be put into each candidate to either validate or confidently reject this match. Candidates entering the final probability scoring stage have a preliminary score greater than the user defined minimum and it is assumed that the correct peptide assignment is in the set. A full set of theoretical ions is available for matching during probability scoring including up to two ammonia and two water losses per fragment. The default setting is to use y, b, and a ions. For parent masses with charge states higher than one, y2+ ions are also generated. The ion series are generated by using the pre-computed y ion series contained within the peptide object. Other theoretical ion types can be generated if desired in the *createFrags *function. New metrics can be inserted in this probability scoring section of the algorithm.

Harvest uses log odds (LOD) scores as the basis of its probability calculations. A LOD score as defined in Harvest is the Log of the odds, where the odds are the probability of this match being correct, given the evidence, over the probability of this match being random (null model), given the same evidence, where random peptides are drawn from a reverse database. Using this definition of a LOD score, a score of zero means that the candidate match is as likely to be correct as it is to be random, with greater positive scores indicating a higher level of confidence that the candidate is a correct assignment (i.e., non-random). One of the advantages of using LOD scores during the probability scoring process is that the LOD scores for separate pieces of knowledge relating to the fragmentation process can be independently integrated into the final score by simple addition. It should be remembered that LOD scoring schemes assume independence of the factors constituting the score, which is unlikely to be strictly true. For this reason, the combination of metrics during scoring may confound interpretation, especially if these features are non-independent. However, using minimally related features of the spectrum during scoring will produce a good approximation.

The use of LOD scores allows the developer to systematically test for the additional discriminating power of new knowledge relating to the fragmentation process by introducing the additional metric into the scoring process. They can then consider the distribution of scores for known incorrect peptides against a set of peptides that have a high probability of being correctly assigned, with an improvement in the separation of these sets being indicative of a good metric. Each piece of knowledge can be leveraged by looking at the features of the unidentified spectrum.

The feature LOD equation can be expressed as:

$$featureLod=Log[\frac{P(correct|E)}{P(random|E)}]$$

where P(correct|E) is the probability of the spectrum under consideration having the specific feature relating to this LOD score, if it was produced by the candidate theoretical sequence, given the evidence E, and P(random|E) is the probability that this feature will have been observed if the spectrum was generated by a random peptide sequence, given the evidence E. In many instances, the differences between metrics will be measured at the fragment ion level, in which case the fragment LOD scores of each of the matching ions is the only relevant information. There are, however, metrics operating at the peptide level, that is, using the aggregate information from the whole peptide. For example, a spectrum dot product between observed and predicted ion intensities, or if considering the effect of non-adjacent amino acids on fragmentation (such as distant basic residues retaining charges). For this reason, Harvest provides a peptide LOD, which, by default, is simply the sum of the individual fragment LOD scores for the peptide:

$$peptideLOD=\sum_{i=0}^{n}featureLODi$$

where i represents a feature (such as a peak match), and n is the total number of these features (such as the number of matched peaks).

Users may ignore the peptide LOD if working with metrics only relevant to fragments. In the case where users choose to apply their own metrics at the peptide level, this default definition of the peptide LOD can be replaced by any other metric operating at the peptide level, using the same interface used to apply metrics operating at the fragment level. The individual fragment LOD scores, plus the peptide LOD score for each candidate, is printed out to a log file, showing information on peak matches, individual peak scores, and whole peptide LOD scores.

### Implementing a novel metric

Harvest is designed for metric development. To demonstrate this utility, we use four metrics, each progressively derived from the previous metric by adding more information. These metrics are simple and not intended to be significant advances in the field. Their purpose is to demonstrate the utility of Harvest for building a non-trivial, experiment specific, data-driven metric in several stages. A metric combining statistical machine learning and neural networks is also described in this paper to demonstrate the application of more complex metrics using Harvest.

It is well established that for any given input sample, different classes of MS/MS machine will produce significantly different output spectra [4,5,7,9,11,14-17,20,22]. In this paper we use Harvest to demonstrate and validate the development of four novel metrics using dataset specific knowledge to improve peptide assignment confidences. First, we demonstrate the implementation of a basic metric for peptide scoring (metric 1). Then we show how the use of experiment specific fragmentation information can be used to build upon this simple metric to improve confidences for correct peptide matches (metrics 2, 3, and 4). This is done in both the gold standard dataset (Aurum) and in the context of a high throughput experiment (Cooksey). Each of these metrics integrates increasingly detailed fragmentation information, to provide increased discriminating power. These metrics are validated against the Aurum dataset and improve upon the number of peptides identified at the 5% false discovery level when compared to X!Tandem. The improvement is seen in both the validated Aurum dataset (MALDI) and the Cooksey dataset: a typical, moderate size non-validated dataset (ESI). Lastly, we demonstrate the use of Harvest to explore physio-chemical properties of fragmentation not related to the peptide identification problem.

The four metrics used to demonstrate the progressive development of a metric using Harvest are:

#### Metric 1: a basic metric

Using this metric, correctly identified fragment matches are assumed to occur with a fixed probability and random peak matches are assumed to occur with an even distribution across the mass range for the peptide file. This metric approximates the scoring metrics used in most protein identification engines in which each fragment "hit" contributes an equal amount to a peptide score. The probability of a correct fragment match for metric 1 is fixed at 0.5. This reflects the fact that approximately half of the peaks under consideration can be identified for correct peptides in this dataset. Without further information, any peak match for a correct assignment has a probability of 0.5. The peaks included in this set are described in the above preliminary and probability scoring sections. The probability of a random match is the probability that a peak would be found within the range between the observed and theoretical peak if the peaks from this spectrum were randomly distributed.

For example, consider the case where the smallest peak in the spectrum is 150 Da, the largest is 1150 Da, and there are 100 peaks in the spectrum. Assuming a random distribution, the density of peaks in the spectrum is 0.1 peaks per Dalton. Now consider a candidate fragment match with a difference between the observed and expected mass of 0.25 Daltons. The probability of a random match with this error (plus or minus this difference) is 0.05. With a fixed correct probability of 0.5, and a random probability of 0.05 for this fragment, the fragment LOD score is therefore 2.3.

In the source code for Harvest, this is the default metric.

#### Metric 2: uses empirically derived fragment mass specific information to derive an estimate of correct assignment

The basic metric (metric 1) assumes a fixed probability of a correct match for all fragments. Considering the distribution of all theoretical fragments, which varies by mass, it may be useful to include this information when scoring. For example, there is only a single correct fragment assignment for each peak irrespective of the fragment mass, however for high fragment masses, there are fewer theoretical fragments which may randomly match. This in turn makes the odds of a correct fragment assignment dependent on the fragment mass. The simplest way to include this information is to empirically determine the probability for correct peptide matches of a peak being matched with respect to its mass. These values can then be used to replace the fixed probability of a correct hit used in the basic metric (metric 1) with the probability for the specific fragment mass. The procedure for determining and using the probabilities of a correct fragment match with respect to mass is described below.

Using the output from Harvest using the basic metric (metric 1) with the default settings, we generated a list of high confidence peptide assignments (Z score > 6). These are assumed to be correct. For 1+ and 2+ parent charges, each peak fragment mass was recorded and marked as either matched or not matched, and grouped into fragment mass bins of 200 Da. The proportion of matched over non matched peaks for each bin gives the probability of a correct match for fragments for each the mass range (bin). Rare 3+ parent ions were given a fixed value (specifically, the average of all the 1+ and 2+ proportions) as their numbers were too low to allow confident modelling. Using these proportions allowed a direct look-up of probability for correct matches during calculation (as per the numerator in the first equation). For example, using the Aurum dataset, it was found that 40% of the fragments between 200 and 400 Da could be identified in correct peptides for this dataset, whereas 80% of the fragments between 1400 and 1600 Da could be identified. Therefore, using this metric, the probability of matching a peak in a correct peptide assignment for a fragment of 300 Da was 0.4. For a fragment of 1500 Da the probability of a correct match was 0.8. A comparison of metric 1 and metric 2 was then carried out for the Aurum dataset.

#### Metric 3: builds upon metric 2 by producing more accurate estimates for random assignments

Having modelled the probability of correct matches for this dataset with respect to fragment mass in the previous metric, we can now apply the same technique for the other half of the equation - the probability of a random match. Instead of assuming an even distribution of random fragments as in the basic metric (metric 1) and the previous metric (metric 2), we add in this metric (metric 3) an empirical estimate of the probability of a random match with respect to the fragment mass. To estimate random fragment matches with respect to mass, the same process of recording each fragment in the spectrum as matched or not matched along with the mass is used (as in metric 2), although this time removing the minimum Z-score requirement and using a reversed peptide database. With the probabilities of fragment matches grouped into bins of 200 Da for both correct (from metric 2) and randomly assigned peptides (included in this metric), we can now use these tables to generate a LOD score. To achieve this, first the values for correct and random probability distributions are normalised so that the probabilities for each bin sum to one for the correct and random distributions. The odds of a peak match for any given fragment mass is the normalised probability value for a correct assignment, over the normalised probability of a peak match for a random fragment for the appropriate mass bin (as shown in the first equation). In this way the LOD score for any matched peptide is now simply a function of its mass. This metric was tested on the Aurum dataset to validate that the additional information using the distribution of random fragment matches by mass improves confidences in correctly matched peptides.

#### Metric 4: including additional information about mass spectrometer specific fragment mass errors to increase discrimination

The fragment mass tolerances set in protein identification algorithms are designed to include as matches fragments which are correctly assigned but not found at the exact theoretical mass expected. These differences between observed and expected masses are introduced by largely stochastic processes in the instrument and are generally considered using a binary function: either a fragment is matched within this range or otherwise, Instead of a "hit" or "no hit" approach, using a fragment mass tolerance parameter, metric 4 explicitly models information about the observed mass error distribution by fitting a probability density function (PDF) to produce a probability that an assigned fragment is correct based on the mass error. It is identical to metric 3, except that when calculating the probability of a correct match, the mass error is input into the PDF to produce a probability that it is correctly assigned. The probability of correct assignment is then multiplied by this value. The mass error distributions for both the validation (Aurum) and experimental (Cooksey) dataset show an approximately normal distribution in mass errors for correctly assigned fragments.

The PDF used the mean and standard deviation of correctly assigned fragment mass errors in bins of 100 Da to produce a single function for each bin, so that each matched fragment used the PDF associated with its mass bin.

This metric was tested on the Aurum dataset for validation and applied to the Cooksey dataset to measure the improvement gained by combining the features present in metric 4 over the basic metric.

#### Implementation of a complex metric

The modelling of peak intensities is another approach to increase discriminatory power in a novel metric [15-17,19]. Many of the factors known to influence peak intensity are high dimensional and non-linear, and are therefore complex metrics. To demonstrate the use of a complex metric in Harvest, we implemented a neural network to predict peak intensities. The neural network consisted of sigmoidal perceptrons with 8 input nodes, 8 hidden nodes, and a single output node. The inputs to the model for predicting fragment intensity were:

1. Ion type: either no fragmentation, iminium, y, a, or b (0,1,2,3,4)

2. Parent charge

3. Fragment charge

4. Length of the parent peptide

5. Length of the fragment divided by the length of the parent peptide

6. Number of H, K, and R residues in the fragment divided by the number of H, K, and R residues in the parent peptide

7. Identity of the amino acid on the N-terminal side of the fragmentation

8. Identity of the amino acid on the C-terminal side of the fragmentation

The identity of amino acid residues were coded as values between 0 and 1, evenly spaced, and in order of increasing hydrophobicity. The output node for training was taken as the log of the peak intensity after normalising the spectrum so that the maximum peak was 100. The testing and training dataset were derived from a 10% subset of the whole dataset to prevent over-fitting (7,800 peptides), with 1,084 high confidence assignments (Z score > 6) from this set used for training. The Flood neural network package was used to model the neural network. Training was for 20 epochs, using a quasi-newton method. During run-time, a vector of attributes was generated for each identified fragment for the neural network input nodes 1 to 8. For each peak, the neural network predicted peak intensity, producing a predicted peak intensity spectrum. Both the predicted and observed intensity spectra were normalised to sum to 100 and an inner dot product generated from the intensities of matching fragments. To generate a Z score for the peak intensity prediction, a series of random predicted peak heights needed to be generated. To create this random set of predicted intensity spectra the intensities of each of the predicted peaks was iteratively assigned to the previous fragment (wrap-around) until the predicted intensity spectrum cycled back to its original position. This technique has the advantage of preserving the distribution of predicted peak heights for random assignments. The Z score for the peak intensity prediction was the number of standard deviations the dot product for the predicted intensity spectrum was, above the mean of the "random" dot products. The peptide-level Z score for peak intensity prediction was added to the Z score for metric 4 to produce a final Z score for each peptide. This complex metric involving a combination of statistically learned attributes, probability density function modelling, and neural network intensity prediction was applied to the Cooksey dataset and compared to metrics 1 and 4 and X!Tandem.

#### The use of Harvest to explore data not related to the peptide identification problem

The fragmentation specific reporting capabilities of Harvest allow the exploration of fragmentation properties. To demonstrate this functionality we used the output of Harvest for the sample set of 500 identified peptides drawn from the Aurum dataset to examine the influence of theoretical fragment isoelectric point (pI) on the presence or absence of y or b ions. In cases where only the y or the b ion was detected, the pIs of the found and missing fragments were calculated. The relative pI of the y ion with respect to the b ion was plotted against the proportion of the length from N to C terminal at which the fragmentation event took place. The use of Harvest in this way provides both a statistical and visual representation of the instrument and experiment dependent fragmentation process.

## Results

### Metric 1

The first 500 identified peptides from the Aurum dataset were used for the comparison of metrics. Metric 1 was used in Harvest to identify peptides at a 95% confidence level (Z score: 3.29). X!Tandem was run with equivalent parameters (modifications, fragment tolerances, etc) and with an expectation value of 0.05. The Harvest and X!Tandem sets were ordered by confidence. Using a 5% false discovery cut-off against the validated peptides in the Aurum dataset, Harvest, using metric 1, correctly identified 461 peptides and X!Tandem correctly identified 464. In this case, there was no significant different in sensitivity between the Harvest basic metric and X!Tandem using the Aurum dataset (p = 0.81). Metric 1 was then used as a benchmark for further development of metrics. For the Cooksey dataset, X!Tandem identified 40,798 peptides using an expectation value of 0.05, while the Harvest basic metric identified 41,929 peptides using a Z score cut off of 3.29.

### Metric 2

**Figure 1:**
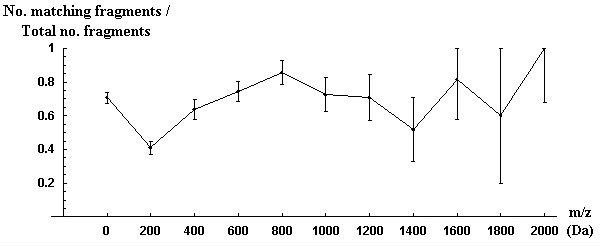
**The proportion of fragments matched (i.e., number of matching fragments divided by total number of fragments) from high confidence peptide assignments as a function of fragment mass for the Aurum dataset**. Error bars show one standard deviation above and below. Fragment mass values are binned into spans of 200 Da, with the first bin containing all fragments <= 200 Da.

Figure 1 shows the proportions of fragments identified for correctly assigned peptides with respect to mass from the Aurum dataset used in metric 2.

**Figure 2:**
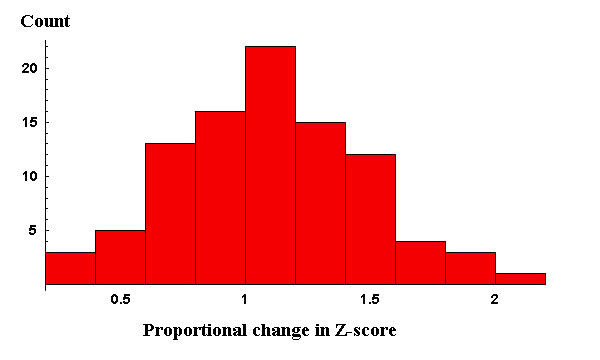
**Histogram of the proportional change in the Z scores of correct peptide assignments when comparing metric 2 to metric 1 using the validated Aurum dataset**. Values greater than 1 represent an increase in the confidence of these peptide assignments.

For the Aurum dataset, the proportional change in the Z scores (confidences) for each correct peptide assignment when comparing metric 2 to metric 1 is shown in Figure 2. In Figure 2, values greater than 1 show an increase in confidence (Z score) for the assignments using metric 2 over metric 1. Values less than 1 show a decrease in confidence. Metric 2 showed an average improvement in the confidence of correct peptide assignments by 11% when compared to metric 1. There was, however, a large variance in "improvement". In this case, it decreases the confidence for many correct peptide assignments, resulting in only a few (2) additional spectra being identified despite the overall increase in confidence for correct assignments. This suggests that this metric should be further developed before use in a protein identification context.

### Metric 3

**Figure 3:**
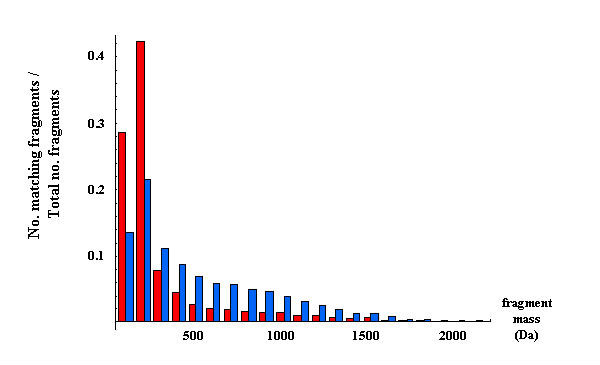
**Proportion of fragments identified (i.e., number of matching fragments divided by total number of fragments) for correctly assigned (blue) and randomly assigned (red) peptides, grouped by fragment mass, for the Aurum dataset**.

Metric 3 improves upon metric 2 by including information about fragment matching probabilities for not just correct, but also random fragment matches. The probabilities of a fragment match for high confidence peptides (Z score > 6) and random assignments with respect to mass for the Aurum dataset is shown in Figure 3.

**Figure 4:**
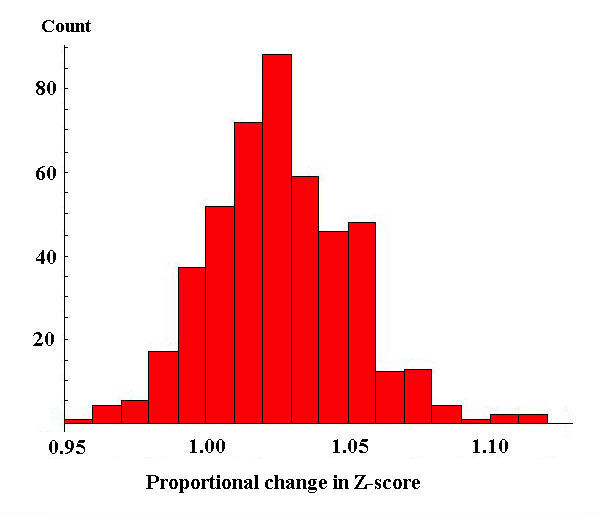
**Proportional change in Z score when using metric 3 compared to metric 1 for the validated Aurum dataset**. Values greater than 1 represent an increase in the confidence of these peptide assignments.

The higher relative values for the correctly assigned fragments (blue bars) above 300 Da in Figure 3 characterise the increased likelihood of a correct match relative to a random match with increasing fragment mass. The use of this additional information in metric 3 gives an average improvement in Z scores for correctly assigned peptides of 2.7% over metric 1, with 86.2% of correct peptide assignments showing increased confidence. Metric 3 improved the number of correct identifications at the 5% level over metric 1 by 7 peptides to 468. Figure 4 shows the change in Z score for correct peptides in the Aurum dataset when comparing metric 3 to metric 1.

### Metric 4

**Figure 5:**
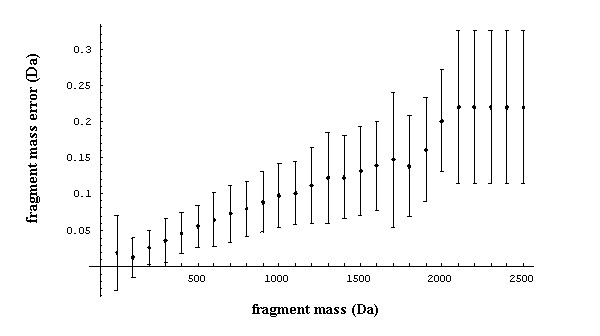
**Mean and standard deviation (error bars) of fragment mass error (observed mass minus expected mass) for correct fragment assignments at various fragment masses for the Aurum dataset**. These values are used to fit probability density functions over correct fragment assignments, with fragment masses grouped into bins of 100 Da.

Metric 4 adds additional information to metric 3 by fitting a fragment mass error probability distribution for correctly matched peptides. This information can be derived from the analysis of any Harvest output. Figure 5 shows the mean fragment mass error and standard deviation for correct peptides across the fragment mass range of the Aurum dataset.

**Figure 6:**
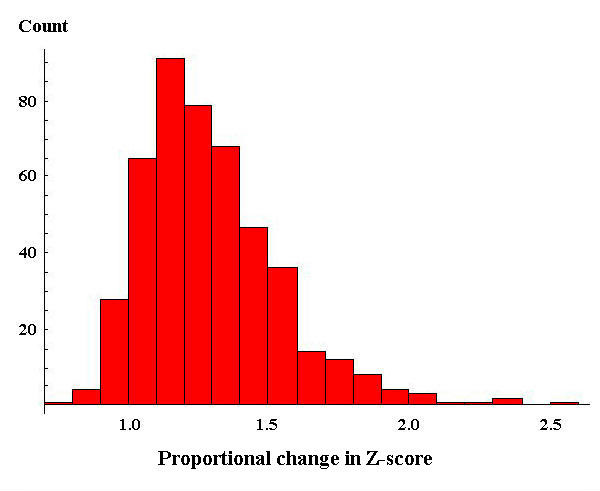
**Proportional change in Z scores using metric 4 when compared to metric 1 for the validated Aurum dataset**. Values over 1 represent an improvement in peptide assignment confidence.

A clear trend is apparent in Figure 5 showing a "drift" with increasing fragment mass between expected and observed fragment masses. Metric 4 uses this information to correct for this drift and to fit a probability density function for each fragment mass bin for correct peptide assignments. The distribution of the change in Z scores for correct peptide assignments using metric 4 compared to metric 1 are shown in Figure 6.

When compared to metric 1, metric 4 increased Z scores for correct peptides for 93% of correct peptide assignments, with an average improvement in confidence (Z score) of 30%. The use of metric 4 increased peptide identifications at the 5% false discovery level by 18 when compared to metric 1. This represents a 3.2% increase in peptide identifications over X!Tandem using this sample set.

After validation, metric 4 was applied to the Cooksey ESI MS dataset of 77,779 spectra. Metric 4 identified 44,412 spectra, an improvement over the basic metric (metric 1) of 2,723 peptides (6.5%), and an improvement over X!Tandem of 3,614 peptides (8.8%).

### Use of a complex novel metric

The use of a neural network to predict peak intensities is an example of a metric operating at the peptide level. The Z scores generated by the neural network based metric were therefore added to (or subtracted from) the peptide LOD score. When the neural network for peak intensity prediction was added to metric 4 and applied to the Cooksey dataset, and additional 78 peptides were identified. The mean increase in Z score for identified peptides was 1.2%.

### Analysis of peptide fragmentation spectra not related to protein identification

**Figure 7:**
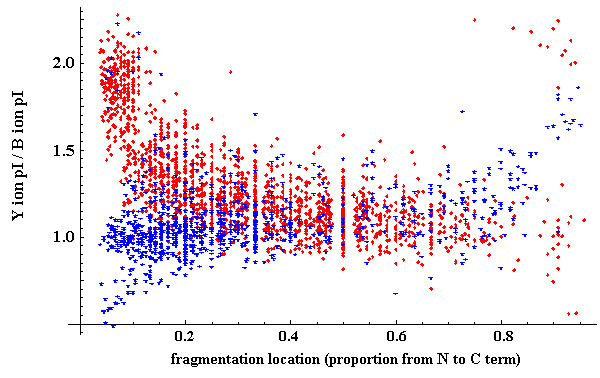
**Relative isoelectric point (pI) of y ions over complementary b ions plotted by location of the fragmentation location between the N and C terminal (proportional)**. Red points show the relative pI of y ions for which the complementary b ion was not detected in the spectrum and the blue points show the relative pI for b ions for which no complementary y ion was found.

Harvest output provides the identities and properties of matched fragments. Using this data, statistical or visual representations of the fragmentation process can be produced. Using the method described above, we show in Figure 7 a comparison of y and b ions (restricted to those without their corresponding ion) plotted against theoretical fragment pI.

## Discussion

Unlike algorithms such as Sequest, Mascot, or X!Tandem [23], Harvest is not designed to extract the maximum number of identified peptides from a dataset, nor is it limited to developing metrics intended for insertion into existing protein identification packages. Rather it is designed to examine hypotheses relating to knowledge of the fragmentation process. The underlying candidate generation processes in common with protein identification algorithms merely serves to provide sets of high confidence assignments to unknown spectra. Insights gained from Harvest may later be used to develop better metrics for protein identification or to use this information without application to the protein identification problem. One example of the use of Harvest without the end goal of developing metrics for protein identification algorithms has been shown in Figure 7. In this experiment, Harvest was used to collect information on identified fragments where their complementary fragments could not be found (y and b ions only). A plot of relative y ion pI with respect to b ion pI over the proportion of the parent peptide along which the fragmentation occurred characterises the inhibitory effects of basic residues for these fragments. The bias towards high pI for shorter y ions (red) is expected as the peptides in the Aurum dataset were all tryptic, however the cluster of long, high pI identified y ions (red), and the cluster of long medium pI y ions (blue), has, to our knowledge, not been previously reported in the literature. It may reflect previously undescribed fragmentation phenomena specific to MALDI datasets.

Other possible hypotheses to be explored include the elucidation of the properties of fragmentation spectra for different machines or experiments, or questions related to steric hindrance, peptide oscillations, or the location of mechanical moments during fragmentation.

As Harvest is specifically geared towards analysis of peptide fragmentation, the outputs of the program include complete lists of the identities and properties of each of the ions matched. This level of reporting is critical for the development of new metrics and for the exploration of the fragmentation processes.

The metrics 1 through 4 demonstrated in this paper are deliberately simplistic and are intended only to demonstrate the way in which any given metric may be assessed and developed using Harvest. Using the Harvest framework, investigators can build a wide range of novel metrics specific to peptide identification in their specific research domain, such as including more information about fragmentation processes, testing physio-chemical hypotheses, or for fine tuning machine learning models.

An exhaustive set of peptide identifications is not always necessary to assess the effect of a new metric for a given dataset. Although, when seeking to test properties of peptides that are not homogenous for all fragments, such as metrics based on the amino acid composition of fragments, many more peptides will be required to validate an improvement.

As the development of metrics is based on the collection of information from the highest confidence peptide assignments, this training set provides the highest confidence information about the experiment specific parameters. An assumption is made that the parameters of this training set apply to the dataset in general. This assumption is reasonable if the training set is representative of the general dataset for the parameters used in the metric. In the case of metrics 1 through 4 presented above, this assumption is reasonable because the probabilities derive from a large number of fragments, from across the full range of the fragment mass spectrum, derived from peptides spanning the full mass range of the general dataset, thus presenting a low risk of over-fitting. Any additional peptides identified using a new metric show an unambiguous improvement in the metric. This is because the information used to used to produce the metric was not drawn from these peptides (since they were not previously identified), and therefore these peptides cannot be an artefact of over fitting. For metrics deriving from data for which the distribution or complexity of the data is larger, such as in the presented complex metric using 8 input attributes, a much larger set of data is required for both feature extraction and to validate the effectiveness of the metric.

Low quality spectra will always remain more difficult to identify and to address with improved metrics. However, Harvest does provide a framework within which metrics may be developed specifically for low quality spectra that have been identified, or for producing metrics better able to identify unknown low quality spectra. This can be achieved because the parameters used in the metrics have been developed specifically for the experimental conditions being investigated.

Harvest provides a simple and flexible platform to enable the user to quickly develop and test new metrics using a set of high confidence peptide identifications drawn from the dataset. The run times required to process a set of peptides is similar to the run times required for protein identification algorithms. However, Harvest allows for a quick turn-around time between hypothesis and test due to the simple interface. By modifying the candidate selection section of the code, users can provide their own parameters for choosing high confidence peptides, or use the default candidate generation process that follows the same basic methods as used in protein identification algorithms. The methods Harvest uses for candidate selection are similar to those used in protein identification packages. This is done so new metrics are developed in an environment closely modelling that into which they may eventually operate.

As metrics for peptide assignment are the fundamental core of protein identification algorithms, any such metrics validated using Harvest are good candidates for insertion into any one of the popular probability based protein identification algorithms to improve their performance or to extract more information from old datasets. The few open source protein identification packages currently available, X!Tandem and OMSSA[23,24], are not readily adaptable to the direct assessment of arbitrary new information to improve peptide matching metrics. For example, the popular protein identification package X!Tandem, provides pluggable scoring to give a greater degree of control during scoring. The X!Tandem pluggable scoring API provides a number of functions which can be overridden or commented out to provide developers access to various parts of the program in order to modify the way in which scoring proceeds. However, there are three significant limitations in the X!Tandem pluggable scoring. Firstly, the user is required to work within the limitations of the scoring pipeline and data structures provided. For example, each peptide must pass through a number of pre-processing steps affecting scoring (prescore, mconvert, hfactor, sfactor, and hconvert) and then through a set of peptide scoring systems (dot, score, and sfactor). Each of these steps require the use of the data structures defined by the X!Tandem spectrum object. The second limitation is that arbitrary use of information cannot be made available during scoring. For example, while the X!Tandem pluggable scoring API provides for extra information to be stored through the 'add_details' function, the standard release is not designed to accommodate complex objects into the spectrum object, such as a machine learning module, support vector machine or neural network. The third limitation is that while it may be possible to implement modifications to the scoring metrics of X!Tandem outside the scope of the pluggable scoring, this would involve an in-depth understanding of the full details of the package. While overcoming these limitations may be possible for an highly experienced programmer, it remains a barrier to other researchers interested in exploring fragmentation properties. Harvest overcomes each of these three limitations by:

1. Not limiting the types of scoring calculations that a user may perform during either fragment or peptide scoring

2. Providing an insertion point at which arbitrary methods, models, or structures may be used during scoring

3. Providing the user with unlimited scope for generation, use, or manipulation of any information relating to either the observed or theoretical spectrum at a single, clearly marked point, along with instructions on how to access this information.

**Figure 8:**
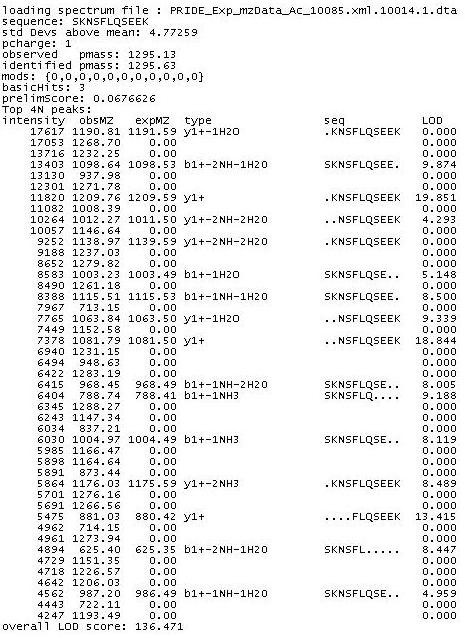
**Sample output for a single identification from Harvest**. Key features such as matching fragments, properties of the parent peptide, source file, and modifications are shown. Fragments are ranked by intensity, with observed (obsMZ) and expected (expMZ) m/z values shown. Expected m/z values and identified parent mass (pmass) are theoretical m/z values. The overall LOD score is the sum of the fragment LODs. Dots in the 'seq' description represent residues not present.

A key advantage of Harvest is that it is able to directly assess peptide-matching metrics for arbitrary scoring functions in a simple stand-alone package, making the process of assessing new metrics simple and efficient. Generally, algorithms incorporating elements in which peptide or protein identification would benefit from optimising metrics based on features of the spectra, may use Harvest as a stand alone tool for the easy implementation and prototyping of metrics based on such features [31,32]. The output of Harvest is a text file with the key features for each peptide identification in a simple, human-readable output as shown in Figure 8. This output is geared towards the intended audience, which include biologists without high-level object-oriented programming skills. For these users, a simple text output with key information for each match makes interpretation of the identifications as easy as possible, and makes the use of popular scripting languages for manipulating results simpler than for XML outputs such as those generated with X!Tandem.

Users of this software may take advantage of its flexibility to develop any number of metrics to improve protein identification algorithms or to explore fragmentation hypotheses independent of the protein identification problem. An obvious example would be to develop a data-driven machine-learning model as a metric used to compare observed and expected fragment peak intensities. Machine learning has previously been introduced in the protein identification domain [15,16,19,33], but interpretation of the effects of various parameter choices is difficult for results reported at the whole protein level. Machine learning algorithms designed specifically to improve peptide identifications such as the Riptide model described by Klammer *et al*. [34], or algorithms such as Percolator [35], could be combined under Harvest with non-machine learning methods to assess the use of a combined approach. Harvest may equally be used to test physio-chemical models [18,30] using a similar process of deriving probabilities given differences in observed and expected fragment peak intensities resulting from the model. The authors plan to use Harvest to assess the utility of new models for MS/MS fragmentation, including machine learning models, analysis of machine specific m/z recording error and models combining vibrational and steric information.

## Conclusions

Harvest bridges the gap between hypothesizing how new knowledge about the fragmentation process may be exploited and using this knowledge to identify more peptides. Furthermore, through the subsequent use of these metrics in external protein identification packages, metrics developed using Harvest may help to identify more proteins. Existing open source software such as X!Tandem [23] and OMSSA [24] are large and complicated programs focused on whole protein identifications rather than on metric validation. As a result, they do not provide a simple way for arbitrary new metrics to be tested on identifications at the peptide level, and as such are limited in their ability to assess new metrics. Harvest provides this functionality by providing a framework for the easy implementation, prototyping, and validation of new metrics as a stand-alone process. The introduction of Harvest will allow researchers to explore specific datasets for exploitable information and assess the utility in general of new metrics for peptide identification, which can then be used to improve protein identification packages. Harvest will be freely available from the Proteome Commons http://proteomecommons.org/.

## Availability and requirements

**Project name: **HARVEST

**Project home page: **https://proteomecommons.org/tool.jsp?i=1039

**Operating system(s): **Tested on PC

**Programming language: **C++

**Other requirements: **None

**License: **FreeBSD

**Any restrictions to use by non-academics: **None

## Authors' contributions

LM wrote the code and acquired the data. Both authors were involved in the conception and design of the study, the analysis and interpretation of data, and the drafting and revision of the manuscript.
